# Influence of Citrus Essential Oils on the Microbiological, Physicochemical and Antioxidant Properties of Primosale Cheese

**DOI:** 10.3390/antiox11102004

**Published:** 2022-10-10

**Authors:** Gabriele Busetta, Marialetizia Ponte, Marcella Barbera, Antonio Alfonzo, Antonino Ioppolo, Giuseppe Maniaci, Rosa Guarcello, Nicola Francesca, Eristanna Palazzolo, Adriana Bonanno, Giancarlo Moschetti, Luca Settanni, Raimondo Gaglio

**Affiliations:** Department of Agricultural, Food and Forest Sciences, University of Palermo, Viale delle Scienze, Bldg. 5, 90128 Palermo, Italy

**Keywords:** ovine cheese, citrus essential oils, *Lactococcus lactis*, physicochemical properties, volatile organic compounds, antioxidant capacity

## Abstract

The aim of this study was to produce a fresh ovine pressed cheese within Pecorino “Primosale” typology with the addition of citrus essential oils (EOs). For this purpose, ewe’s pasteurized milk was added with EOs from the peel of lemons, oranges and tangerines. Seven cheese productions were performed at the pilot plant scale level, including one control production (CP) without the addition of EOs and six experimental productions obtained by the addition of two EO concentrations (100 and 200 µL/L) to milk. The acidification process was obtained by means of the starter cultures *Lactococcus lactis* CAG4 and PON36. All cheeses showed levels of lactic acid bacteria (LAB) around 10^9^ CFU/g, indicating that citrus EOs did not negatively influence the starter evolution. The addition of citrus EOs did not determine significant variations for dry matter, fat and protein percentages but increased the antioxidant capacity of all the experimental cheeses of about 50% in comparison to the control trial. The citrus EOs impacted cheese VOCs, especially for terpene class (limonene, β-pinene, myrcene, carene, linalool and α-terpineol). The sensory evaluation showed that cheeses enriched with 100 µL/L of citrus EOs were mostly appreciated by the panelists.

## 1. Introduction

In the past few years, the negative consumers’ acceptance for synthetic chemical preservatives determined an increasing interest in natural alternative preservatives to produce food with long shelf-lives, high nutritional values and sensory characteristics [1]. Therefore, food industries and academic researchers focused their attention on the use of active compounds derived from plant and fruit byproducts [2]. This strategy fits well within the European Green Deal aimed at highlighting environmental sustainability through the reuse of food wastes of processing industries for a clean and circular economy [3]. In this context, particular attention has been paid to plant essential oils (EOs) obtained by steam distillation; dry distillation or a suitable mechanical process of different parts of the plants, including the leaves, flowers, peels, barks, seeds and roots [4].

EOs are a mixture of secondary bioactive metabolites, such as phenylpropenes, terpenes and other volatile constituents [5]. These substances are natural aromatic and volatile liquids known since ancient times for their flavoring properties [6] and antimicrobial properties [7]. Nowadays, the positive effects of EOs to exert antioxidant, anti-inflammatory and antispasmodic actions are well-known [8]. Although some essential oils may cause risk to human health [9], most of them are harmlessness and classified as Generally Recognized As Safe (GRAS) by the U.S. Food and Drug Administration, and their use is allowed in all foods [10]. Among the raw plant materials used for the extraction of EOs, the byproducts of the citrus industry have been extensively considered [11].

The region of Sicily is a leader in the cultivation of several citrus genotypes (pummelo, grapefruit, orange, kumquat, mandarin and lemon) [12], and their fruits are consumed fresh or processed into juices and candied items [13]. The main waste generated during the processing of fruits is represented by epicarp or flavedo [14], unexploited byproducts in juice production [15]. However, this byproduct contains numerous oil glands filled with EOs [16], which have a wide spectrum of applications, including pharmaceutical, cosmetic, agricultural and food formulations [17].

The use of citrus EOs in dairy production is not new; so far, this application has only been performed for bovine milk-derived cheeses in order to improve their microbiological stability [18,19]. To our knowledge, no studies regarding the addition of citrus EOs to ewes’ milk cheeses are present in the literature. Generally, Sicilian fresh-pressed cheese made from ewes’ milk are referred to as Pecorino “Primosale” cheeses [20], which are commonly consumed after a very short ripening time [21].

The purpose of the present research was to produce, for the first time, a new typology of Pecorino “Primosale” cheese with the addition of three industrial citrus EOs extracted from oranges, lemons and tangerines and *Lactococcus lactis* starter cultures. The specific objectives of the present study were to: (i) evaluate the in vitro inhibitory activity of citrus EOs against starter cultures; (ii) characterize the citrus EOs for their chemical compositions; (iii) monitor the ability of starter cultures to drive the fermentation process in the presence of citrus Eos and (iv) characterize the final cheeses in terms of their physicochemical traits, antioxidant capacity, volatile organic compounds and sensory characteristics.

## 2. Materials and Methods

### 2.1. Raw Materials and Starter Cultures

Three industrial citrus EOs extracted by cold pressing from the peels of oranges (*Citrus sinensis* (L.) Osbeck), lemons (*Citrus limon* (L.) Osbeck) and tangerines (*Citrus reticulata* Blanco) were provided by the “EuroFood S.r.l.” facility located in Capo d’Orlando (Messina, Italy). Whole ewes’ milk used for cheese production was obtained exclusively from Valle del Belìce breed sheep. Two strains of *Lactococcus lactis* subsp. *lactis* (CAG4 and PON36) belonging to the culture collection of the Department of Agricultural, Food and Forest Sciences (University of Palermo, Italy), previously isolated from raw ewes’ cheese productions and evaluated for their dairy traits [22,23], were used as the fermenting agents.

### 2.2. Inhibitory Assay

In order to evaluate the suitability of citrus EOs to produce Primosale cheese, their inhibitory activity was tested against the two *Lc. lactis* starter strains. Following the methodology described by Gaglio et al. [24], citrus EOs were tested against a cell density of approximately 10^7^ colony-forming units (CFU)/mL of each *Lc. lactis* strain in Media 17 (M17) soft agar (0.7% *w/v*) (Biotec, Grosseto, Italy) applying the paper disc diffusion method. Sterile water was used as the negative control, while streptomycin (10% *w/v*) as the positive control [25]. The inhibitory activity was evaluated after incubation at 30 °C for 24 h and was scored positive only in the case of a definite clear area surrounding the paper discs. This test was carried out in duplicate.

### 2.3. Development of Natural Milk Starter Culture

The Natural Milk Starter Culture (NMSC) was prepared according to Gaglio et al. [26]. Briefly, the strains *Lc. lactis* CAG4 and PON36 were reactivated in M17 broth (Biotec) incubated at 30 °C for 24 h. After growth, the cells were centrifuged at 10,000× *g* for 5 min, washed and resuspended in Ringer’s solution (Sigma-Aldrich, Milan, Italy). Washed cells were then inoculated into ovine whole-fat UHT milk (Leeb Vital, Wartberg an der Krems, Austria) at a final concentration of about 10^6^ CFU/mL. After incubation at 30 °C for 24 h, the NMSC containing the multi-strain culture was used for cheese making.

### 2.4. Cheese Productions and Sample Collection

The experimental cheese productions were performed under controlled conditions at a dairy pilot plant of the dairy factory “Biopek”, located in Gibellina (Trapani, Italy). The experimental plan included a control production (CP) and two experimental productions (EP), one for each concentration of citrus EOs (100 and 200 µL/L) added to ewes’ milk; the EO concentrations were chosen based on the previous study of Marcial et al. [27]. A total of seven productions were planned (Table 1).

**Figure 1:**
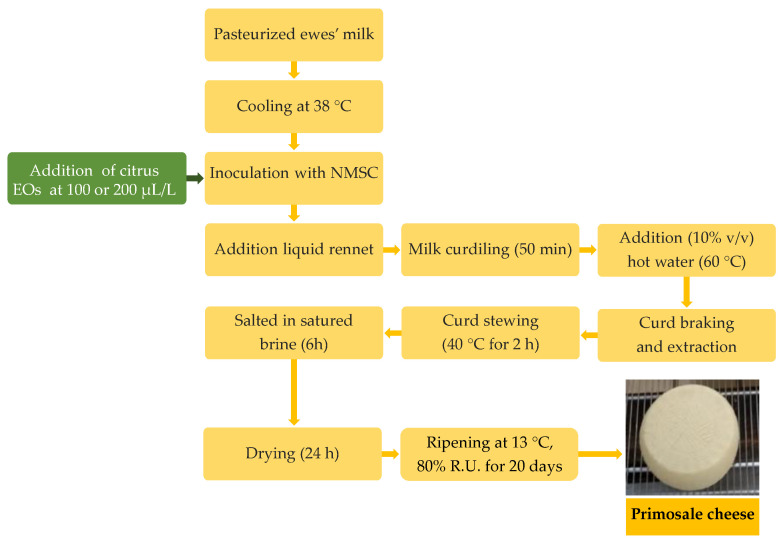
Flow diagrams of Primosale cheese production enriched with citrus essential oils. Abbreviations: EOs, essential oils; NMSC, natural milk starter cultures.

Cheese production was performed by applying the technology for the “Primosale” pressed cheese type (Figure 1).

Briefly, each trial was performed in plastic vats with 25 L pasteurized (72 °C for 15 s) whole ewe’s milk. After cooling at 38 °C, the bulk milk was inoculated with the NMSC (250 mL) and added to 100 or 200 µL/L of each citrus EOs prior the liquid rennet (Clerici Sacco International, Cadorago, Italy) addition (3 mL). After coagulation, the curd was cut to the dimension of rice grains, hand-pressed into cylindrical, perforated plastic molds and subjected to stewing at 40 °C for 2 h. The cheeses were salted in saturated brine for 6 h and ripened for 20 d in a storage chamber at 13 °C and 80% relative humidity (RH). Cheese productions were carried out in duplicate in two consecutive weeks. Samples of raw milk, pasteurized milk, inoculated milk, curds and the final cheeses were collected for analysis.

### 2.5. Microbiological Analyses

Milk samples were directly serially diluted (1:10) in Ringer’s solution (Sigma-Aldrich, Milan, Italy) into 10-mL/volume test tubes, while the curd and cheese samples (10 g) were first, homogenized in sodium citrate (2% *w/v*) solution by means of BagMixer^®^ 400 (Interscience, Saint Nom, France) at the highest speed for 2 min and then serially diluted in Ringer’s solution. The dilutions of raw milk and pasteurized milk were plated on agar media to analyze the total mesophilic microorganisms (TMM), and the main pro-technological (lactic acid bacteria (LAB), including enterococci); spoilage (*Pseudomonas* spp.) and pathogenic (members of the Enterobacteriaceae family, coagulase-positive staphylococci (CPS), *Listeria monocytogenes*, *Escherichia coli* and *Salmonella* spp.) bacterial groups following the approach of Gaglio et al. [28].

### 2.6. Isolation, Typing and Identification of LAB Resistant to the Pasteurization Process

After growth, all colonies of presumptive LAB sharing different morphologies in terms of the shape, size, color, edge, characteristics of the surface and elevation were isolated from the highest cell densities of pasteurized bulk milk. All isolates were preliminary characterized by Gram determination applying the method described by Gregersen [29] and catalase test, determined as reported by Koneman et al. [30]. Only Gram-positive cultures negative for catalase expression were purified by consecutive subculturing and differentiated by randomly amplified polymorphic DNA (RAPD)-PCR analysis applying the protocol described by Gaglio et al. [31]. Gelcompare II software version 6.5 (Applied-Maths, Sint-Martens-Latem, Belgium) was used to analyze the resulting RAPD profiles. All different strains were identified at the species level by 16S rRNA gene sequencing following the approach applied by Gaglio et al. [32]. The sequences were compared with those available in the GenBank/EMBL/DDBJ (http://www.ncbi.nlm.nih.gov, accessed on 14 September 2022) [33] and EzTaxon-e (http://eztaxon-e.ezbiocloud.net/, accessed on 14 September 2022) databases [34].

### 2.7. Starter Cultures Recognition

All presumptive LAB collected during cheese making from inoculated milk with NMSC until the final cheeses were analyzed at the strain level by means of RAPD-PCR in order to monitor the dominance of lactococci inoculated as starter cultures (*Lc. lactis* CAG4 and PON36) over LAB resistant to pasteurization.

### 2.8. Physicochemical Analysis of Cheeses

Cheeses were sampled and evaluated for their physicochemical traits. Cheeses were assessed for external and internal colors, measured in duplicate by a Minolta Chroma Meter CR-300 (Minolta, Osaka, Japan) using the illuminant C. The results are expressed as lightness (L*, from 0 = black to 100 = white), redness (a*, from green = −a to red = +a) and yellowness (b*, from blue = −b to yellow = +b), according to the CIE L*a*b* system [35].

Cheese hardness was evaluated with an Instron 5564 tester (Instron, Trezzano sul Naviglio, Milan, Italy) measuring the maximum resistance to compression (compressive stress, N/mm^2^) of samples (2 cm × 2 cm × 2 cm) kept at room temperature (22 °C).

Water activity (a_w_) was determined by a HygroPalm water activity indicator (Rotronic, Bassersdrof, Germany), according to ISO 21807 [36].

The cheese samples were freeze-dried according to the method reported by Rashidinejad et al. [37] and analyzed for the contents of dry matter (DM) [38], fat [39] and ash [40], whereas protein (% DM) was calculated by difference (100—fat % DM—ash % DM).

### 2.9. Antioxidant Capacity of Cheeses

Cheese samples were analyzed in duplicate for their antioxidant properties, determining the total polyphenols and the Trolox equivalent antioxidant capacity (TEAC).

Total polyphenols were determined by the Folin–Ciocalteau colorimetric method [41], with gallic acid as the standard. Briefly, 100 µL of extracted sample were mixed with 900 µL of distilled water and 500 µL of Folin–Ciocalteau reagent diluted in distilled water to a concentration of 1 N. Then, 2.5 mL of a 20% (*w/v*) sodium carbonate aqueous solution were added, and the mixture was vortexed for 30 s and incubated for 40 min in the dark at room temperature. The absorbance of the samples was read at 725 nm with the HUCH DR3900 spectrophotometer. Aqueous solutions of gallic acid with different concentrations (0–1 mg/mL) were used for the calibration curve (R_2_ = 0.99). The results were expressed as gallic acid equivalents (g GAE/kg DM).

The TEAC, a discoloration test measuring the radical scavenging ability of samples using ABTS radical cation (ABTS • +) and Trolox as the standard [42], was performed to evaluate the antioxidant activity of cheese extracts as described by Bonanno et al. [43], with some modifications. Briefly, the radical cation ABTS was obtained by reacting a 14 mM ABTS aqueous solution with an equal volume of 4.9 mM potassium persulfate and incubating the mixture for 16 h in the dark at room temperature before use. For the assay, the ABTS solution was diluted with 5 mM phosphate-buffered saline (PBS) (pH 7.4) to an absorbance of 0.795 (±0.02) at 734 nm. The absorbance of a mixture of 10.5 µL of PBS with 1.5 mL of a diluted ABTS solution was read at 734 nm after incubation for 6 min at 30 °C. In the same way, 10.5 µL of each extracted sample were mixed with 1.5 mL diluted ABTS radical cation solution, and the absorbance read at 734 nm after incubation at 30 °C for 6 min was used to calculate the percentage decrease of the absorbance due to decolorization in comparison with the absorbance read with PBS. A Trolox solution in PBS, between 0 and 2.5 mM, was used to develop a calibration curve (R_2_ = 0.99), and the results were expressed as mmol Trolox/kg DM.

The oxidative stability of cheese fat was evaluated by determining in duplicate the peroxide value (POV, mEq O_2_/kg fat) as the index of primary lipid oxidation [44], and the thiobarbituric acid-reactive substances (TBARs) as products of secondary lipid oxidation expressed as μg of malonylaldehyde (MDA)/kg DM, according to the methods proposed by Tarladgis et al. [45] and modified by Mele et al. [46]. Briefly, 2 g of freeze-dried cheese were mixed with 8 mL of phosphate buffer aqueous solution (pH 7) and homogenized by an Art-Miccra D-8 high-speed homogenizer (Art Labortechnik, Heitersheim, Germany). After the addition of 30% (*v/v*) trichloroacetic acid aqueous solution (2 mL), the sample was vortexed for a few seconds and filtered with Whatman No. 1 filter paper. An aqueous solution of 0.02 M thiobarbituric acid (5 mL) was added to 5 mL of filtrate, placed in a hot water bath at 90 °C for 20 min and then refrigerated. After centrifugation at 4500 rpm for 5 min, the absorbance of the supernatant was read at 530 nm using a Hach DR/4000 U spectrophotometer. To quantify TBARs, 1,1,3,3-tetramethoxypropane solutions at concentrations between 0.016 and 0.165 µg/mL were used for the calibration curve (R_2_ = 0.99).

### 2.10. Volatile Organic Compounds

Volatile organic compositions of the citrus EOs and cheese samples (CP and EP) were performed by GC/MS analysis. The citrus essential oils were directly diluted with hexane (1:100 ratio), while the cheese samples (2 g) were finely chopped. All samples were placed in a glass vial for the headspace solid-phase microextraction (SPME). SPME (DVB/CAR/PDMS, 50 mm, Supelco, Bellefonte, PA, USA) fiber was exposed to the samples under continuous stirring at 60 °C for 15 min. After sampling, the volatile components were desorbed for 1 min at 250 °C through a GC split-less injector with a SPME inlet liner. GC analyses were performed on an Agilent 6890 gas chromatograph coupled with a mass selective detector (Agilent 5975 c) using a DB-624 capillary column (Agilent Technologies, Santa Clara, CA, USA, 60 m, 0.25 mm, 1.40 µm). Helium was used as the carrier gas with an ionization voltage of 70 eV at a flow rate of 1 mL/min. The oven temperature program was 5 min isotherm at 40 °C, followed by a linear temperature increase of 5 °C min up to 200 °C, where it was held for 2 min. The transfer line temperature was 230 °C, and the scanning range of the GC/MS was 40–400 amu. The volatile components of the samples were identified by comparing their mass spectra to the NIST Library and confirmed by comparison with the Kovat’s Index.

### 2.11. Sensory Evaluation

Sensory evaluation of CP and EP cheeses was performed by a descriptive panel of 15 assessors (eight women and seven men aged between 20 and 62 years old). The judges were trained specifically following the ISO 8589 [47] indications and were asked to score 16 descriptors, including color, structure uniformity, intensity of odor, unpleasant odor, intensity of aroma, salty, sweet, acid, bitter, spicy, unpleasant aroma, adhesiveness, hardness, humidity, taste persistency and overall acceptability. The assessors scored the level of each attribute with a mark on a 7-point hedonic scale (0 = extremely low; 7 = extremely high).

### 2.12. Statistical Analyses

One-Way Analysis of Variance (ANOVA) was applied to identify the differences among the microbiological and physicochemical data. Tukey’s test was applied for pairwise and multiple mean comparisons (statistical significance *p* < 0.05). The values of VOCs emitted from citrus EOs were graphically represent as a heat map generated using ascendant hierarchical clustering. The VOC concentrations were graphically represented by a color change from yellow (lowest concentration) to red (highest concentration). Two-factor analysis of variance (ANOVA), with judges (i = 1, ..., 15) and cheeses (j = 1, ..., 7) as the fixed factors, was applied to evaluate the discrimination efficiency of the sensory attributes for each panelist. *t*-test with significance levels of *p* < 0.05 was used to compare the least square means (LSM). Statistical processing of microbiological and sensory data, as well as the graphic constructions of VOCs emitted from citrus EOs was performed with XLStat software version 2020.3.1 for Excel (Addinsoft, New York, NY, USA), while the physicochemical data were analyzed using the generalized linear model (GLM) procedure in SAS 9.2 software (SAS Institute Inc., Campus Drive Cary, NC, USA).

## 3. Results and Discussion

### 3.1. Suitability of Citrus EOs for Cheese Production

The antimicrobial activity of EOs obtained from the citrus genotypes (oranges, lemons and tangerines) cultivated in Sicily against the main human pathogens, including *E. coli*, *L. monocytogenes*, *Salmonella* spp. and *Staphylococcus aureus* bacteria, is well-known [48,49]. In our work, we tested the three industrial citrus EOs extracted from oranges, lemons and tangerines against LAB, which are necessary to drive the fermentation process and to transform milk into cheese [50]. The citrus EOs tested did not inhibit the growth of the two strains of *Lc. lactis* (CAG4 and PON36) selected as starter cultures (results not shown). Thus, these results confirmed the suitability of citrus EOs for Primosale cheese production, because their presence does not interfere with *Lactococcus* development.

### 3.2. Evolution of Microbial Population during Cheese Making

**Figure 2:**
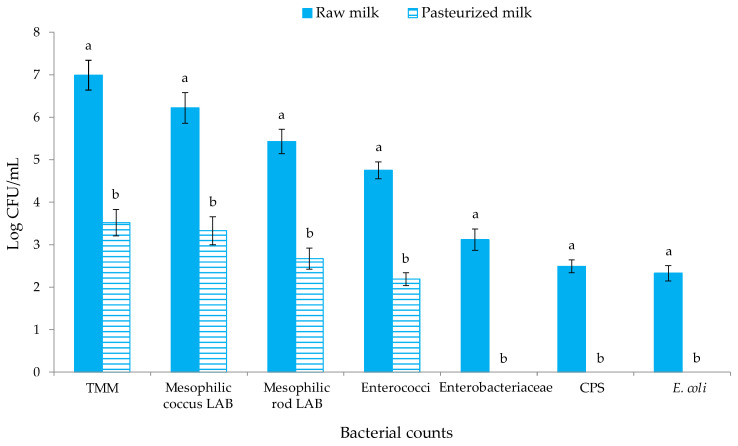
Microbial counts (Log CFU/mL) of raw and pasteurized milk samples. Abbreviations: TMM, total mesophilic microorganisms; CPS, coagulase-positive staphylococci; *E.*, *Escherichia*. The results indicate the mean values and standard deviation of four plate counts (carried out in duplicate for two independent productions). Different superscript letters indicate significant differences in the microbial concentrations according to Tukey’s test between the raw and pasteurized milk samples for *p* < 0.05.

The levels of the different microbial groups investigated in ewes’ milk before and after pasteurization are reported in Figure 2. The presence of *Salmonella* spp. and *L. monocytogenes* was never found (for this reason, these results are not reported in Figure 2).

Statistically significant differences (*p* < 0.05) were observed among the raw and pasteurized milk for the levels of all the microbial group objects of investigation. Ewes’ milk before pasteurization hosted levels of TMM of 6.99 CFU/mL, which is higher than the European limit of <500.000 CFU/mL for raw ewes’ milk [51]. High levels of TMM are often detected in raw ewe’s milk from the Valle del Belìce breed [52,53], and this is imputable to the microbial contamination of the udder surface occurring during the milking procedures or during transport or to the growth of indigenous milk microorganisms during storage [54]. Mesophilic coccus LAB were found at 10^6^ CFU/mL, while mesophilic rod LAB were one Log cycle lower. After pasteurization, the levels of TMM, mesophilic coccus, rod LAB and enterococci, decreased by about three Log cycles, showing the ability of some indigenous milk bacteria to survive until the pasteurization process [55,56]. The levels of undesired microbial groups, such as members of the Enterobacteriaceae family, CPS and *E. coli* in raw milk, were in the range 10^2^–10^3^ CFU/mL and decreased below the detection limit in pasteurized milk. A similar trend was previously observed for ewes’ milk by Barbaccia et al. [57,58]. The analysis of milk after each citrus EO and NMSC addition showed levels of mesophilic coccus LAB above 7 Log CFU/mL, confirming that *Lc. lactis* inoculums occurred at 10^7^ CFU/mL.

Table 2 reports the levels of TMM and *Lc. lactis*, added as a starter culture, on curds and cheeses samples.

After curdling, TMM and *Lc. lactis* were counted at 10^8^ CFU/g, while, in the final control and experimental cheeses, their levels were above 9.0 Log CFU/g. A similar trend was previously reported by Marcial et al. [27] for bovine pressed cheeses produced with an oregano EO addition and using selected LAB strains as fermenting agents. These results highlighted that the addition of citrus EOs did not alter the microbiological parameters of the final cheeses.

### 3.3. Composition of Thermoduric LAB Populations

**Figure 3:**
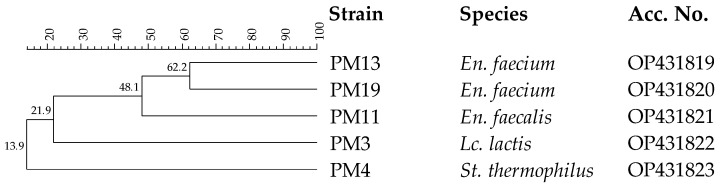
Dendrogram obtained with combined RAPD-PCR patterns generated with three primers for thermoduric indigenous milk LAB strains isolated from pasteurized milk. Abbreviations: *En.*, *Enterococcus*; *Lc.*, *Lactococcus*; *St.*, *Streptococcus*.

Thirty-three isolates from pasteurized milk were considered presumptive LAB, being Gram-positive and catalase-negative. After purification, all isolates were analyzed by RAPD-PCR in order to distinguish the different strains. The dendrogram reported in Figure 3 shows five different strains.

These strains resistant to the pasteurization process were analyzed by 16S rRNA gene sequencing. This analysis confirmed their inclusion in the LAB group, since they were identified as *Enterococcus faecalis*, *Enterococcus faecium*, *Lc. lactis* and *Streptococcus thermophilus*. These species are typical of dairy environments [48]. In particular, *Lc. lactis* and *St. thermophilus* are components of the starter LAB (SLAB) responsible for lactic fermentation, while *En. faecalis* and *En. faecium* to the non-starter LAB (NSLAB) community associated with the development of typical sensory traits [59]. The ability of *En. faecalis*, *En. faecium* and *St. thermophilus* to survive the pasteurization process is well-known [60,61], while *Lc. lactis* is a species sensitive to heat treatments [62], and for this reason, its presence in pasteurized ewes’ milk could be imputable to post-pasteurization contamination [63].

### 3.4. Persistence of the Added Starter LAB Strains

The dominance of *Lc. lactis* CAG4 and PON36 during cheese making was evaluated by means of RAPD profile comparisons. This technique, commonly used to monitor the LAB starter cultures in dairy production [64,65], showed the dominance of the added strains over those that survived the thermal treatment both in the control and experimental productions, excluding any negative influence of EOs.

### 3.5. Physicochemical Characterisation of Cheeses

The physical properties and the chemical compositions of the cheeses are reported in Table 3.

The effect of production was statistically significant for all cheese color indexes. Indeed, the external and internal surfaces of cheeses performed with lemon and tangerine EOs at both levels showed lower values of lightness (L*) and redness (a*) and a corresponding higher yellow index (b*) in comparison with the other cheeses. This effect is presumably related to the transfer of a larger amount of carotenoid pigments that, among those isolable from the respective EOs [66], were able to induce a higher yellow color. Overall, the indexes of internal colors recorded in CP cheeses were within the ranges observed for Primosale cheeses [26,43].

The hardness of the pastes was comparable among the cheeses from different productions, apart from the highest consistency recorded for the cheeses of EPO100 production, which differed significantly from the EPT100 cheeses. No explanation based on variations in chemical components, as increasing the humidity or fat reduction, was found to justify this increase; however, the major consistency of the EPO100 cheeses corresponded to their tendency towards a higher adhesiveness perceived at the sensory level.

The chemical composition of the cheeses in terms of the DM, fat and protein, as well as aw, were not affected by the addition of citrus EOs. Indeed, a slightly higher ash content emerged in EPO200 cheeses, with a significant difference in EPT200 cheeses, which can be attributed to the major ash content of orange EOs or to a greater amount of salt absorbed by cheeses. Additionally, the chemical components of the Primosale cheeses ranged into the levels found by other investigations [26,67,68].

### 3.6. Antioxidant Capacity of Cheeses

The results of the antioxidant capacity of the cheese analyses are reported in Table 4.

As expected, the cheeses from all the experimental productions displayed a higher antioxidant capacity, expressed as TEAC, than the control cheeses, without differences between the Eos and their addition levels. This result confirmed the well-known antioxidant properties of citrus EOs [69], mainly due to their components such as terpenes and phenolic compounds. For this reason, citrus EOs are commonly incorporated into foods to prevent oxidation, to increase the shelf life and to provide health benefits [70]. However, the total polyphenols content did not differ significantly among the experimental and control productions. Thus, the improvement of the antioxidant activity of cheeses fortified with citrus EOs is mainly attributable to other antioxidant molecules, especially terpenes. Furthermore, the antioxidant activity of Eos also derives from the synergic actions of various molecules [71]. Although limonene was the major terpene in all the experimental cheeses, its antioxidant contribution is reported to be limited [72].

Although the inclusion of Eos was able to improve the antioxidant capacity of the experimental cheeses, it was not effective in preserving the cheese fat from oxidation. Indeed, the primary lipid oxidation, expressed as the peroxide value, was lower only in the cheeses fortified with lemon EOs, whereas TBARS, indicating the secondary lipid oxidation, was higher in the experimental cheeses than in the control cheese, although the orange and tangerine EOs induced a better protection from oxidation when used at the highest level. Based on these results, the potential of citrus EOs as sources of antioxidant compounds to preserve cheese fat from oxidation needs to be further investigated, while also considering the maintenance of oxidative stability during the storage time.

### 3.7. Volatile Organic Compounds Emitted from Citrus EOs and Cheeses

**Figure 4:**
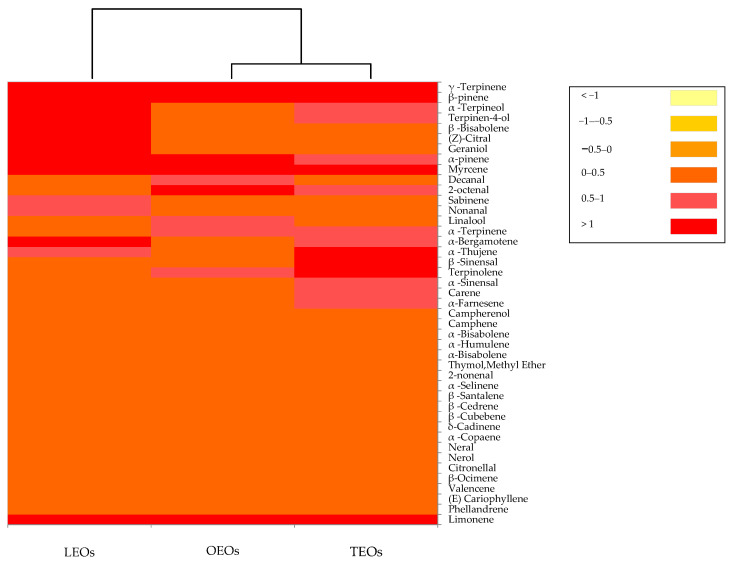
Distribution of the volatile organic compounds among the citrus EOs. The heat map plot depicts the relative concentration of each VOC. Abbreviations: LEOs, lemon essential oils; OEOs, orange essential oils; TEOs, tangerine essential oils.

The volatile profiles generated by the citrus EOs are reported in Figure 4.

The dendrogram resulting from the cluster analysis and the heat map showed the formation of two clusters. The orange and tangerine EOs clustered together into one main group, while the lemon EOs were in a separate single cluster. In particular, in lemon, orange and tangerine Eos, a total of 32, 31 and 26 volatile compounds were identified, respectively. Three phytochemical groups, including monoterpene hydrocarbons, oxygenated monoterpenes and sesquiterpene, were recognized in all three citrus EOs. The monoterpene hydrocarbons constitute the most representative group of volatile compounds, with 94% in the orange EOs, 92% in the tangerine Eos and 87% in the lemon EOs. In all three EOs, the most quantitatively relevant compounds were limonene, β-pinene and γ-terpinene. Limonene, the major compound of the present study, is a cyclic monoterpene with a lemon-like odor and is considered to be the major chemical constituents in various EOs of citrus species [73,74]. In addition to limonene, all three citrus EOs were characterized by the presence of linalool and citral. These components are reported to exert an antimicrobial activity towards bacteria and fungi [75,76]. Basically, the volatile compounds identified in citrus EOs analyzed in this study were similar to those found by other authors [74,77].

Table 5 reports the VOCs emitted from cheeses sample with and without EO additions. The control cheese profile was characterized by 20 compounds belonging to the classes of acids, ketones, aldehydes, alcohols and monoterpene. The main acids identified were hexanoic, butyric and 2-hydroxy4-methyl-pentanoic acids. These compounds contribute to the formation of cheese flavor both directly and indirectly as precursors of odor-active compounds such as ketones and aldehydes [78,79]. Hexenal and heptanal were the main aldehydes detected in the control cheese, and the main alcohol was 1-butanol-3-methyl. Similar volatile compound profiles were also observed in other cheeses produced from sheep’s milk [26,53,80]. The cheeses processed with citrus EO additions emitted 24 VOCs in the presence of lemon EOs and 23 in the presence of orange, as well as tangerine, EOs. Cheeses produced with citrus EOs differed from the control cheeses, especially for the limonene, β-pinene, myrcene, carene, linalool and α-terpineol concentrations. No statistically significant differences were found among the experimental cheeses produced with the addition of 100 and 200 µL/L of citrus EOs. The most abundant VOC found in all the enriched cheeses with citrus EOs was limonene, which showed a slight increase with the increasing percentage of citrus EOs added to milk. The only terpene found both in the control and experimental cheese is α-pinene, commonly present in cheese as a result of pasture feeding [81,82]. The monoterpene hydrocarbons constitute the most representative group of VOCs in experimental cheeses. In particular, in cheeses enriched with 100 and 200 µL/L of lemon, orange and tangerine Eos, monoterpene hydrocarbons accounted for 82%, 83% and 85%, respectively. This class of compounds is commonly found in cheeses enriched with different types of essential oils [83,84]. The higher carryovers observed for monoterpene hydrocarbons with respect to the oxygenated compounds may be explained by possible interactions with fat, carbohydrate and protein matrices in cheese [85,86,87]. Specifically, hydroxyl groups can interact with the receptor groups of proteins, such as NH and CO, determining the higher retention rate for oxygenated compounds in the food matrix, restricting their ability to be transferred [88].

### 3.8. Sensory Aspects of Cheeses

The results of the sensory evaluation are reported in Table 6. This evaluation is commonly used to determine the tasters’ acceptance of a new food product before marketing [89].

In this study, this analysis showed that the addition of citrus EOs led to the production of Primosale cheeses characterized by a sensory profile not particularly different from that generally recognized for traditional Primosale cheeses. In particular, except for the intensity of odor, intensity of aroma and taste persistency that were scored different for the cheeses, all other sensory traits evaluated were not influenced by the addition of citrus EOs. In detail, the intensity of odor and of aroma and taste persistency increased with the concentration of citrus EOs added, confirming the previous findings of Marcial et al. [27], who tested different concentrations of oregano EO to fortify a traditional Argentinean fresh bovine cheese. Although it is well-known the EOs have a strong flavor that can alter the sensory acceptability of dairy products [90], our results highlighted that the cheeses produced with 100 µL/L of citrus EOs were the most appreciated ones by the panelists.

## 4. Conclusions

This study provided an analysis of the microbiological, physicochemical and sensory characteristics of a Primosale cheese processed with citrus EOs extracted from peels of lemons, oranges and tangerines. The enrichment of milk at 100 and 200 µL/L did not negatively influence the fermentation activity of the two *Lc. lactis* (CAG4 and PON36) used as the starter cultures. The addition of citrus EOs to milk did not affect the chemical compositions of the Primosale cheeses while determining a relevant increase of the antioxidant capacity. The volatile profiles of the cheeses enriched with citrus EOs were impacted with monoterpene hydrocarbons and oxygenated monoterpenes compounds. The sensory evaluation indicated that Primosale cheeses enriched with 100 µL/L of lemon, orange and tangerine EOs were characterized by the highest overall acceptability. These results clearly highlighted that the addition of citrus EOs to milk determined the production of novel fresh cheeses able to enlarge the Sicilian ewes’ milk dairy products portfolio.

## Figures and Tables

**Table 1:** Cheese productions.

Trials	EOs	Concentration (µL/L)
CP	-	-
EPL100	lemon	100
EPL200	lemon	200
EPO100	orange	100
EPO200	orange	200
EPT100	tangerine	100
EPT200	tangerine	200

Abbreviations: EOs, essential oils; CP, control productions; EPL, experimental production with lemon; EPO, experimental production with orange; EPT, experimental production with tangerine.

**Table 2:** Growth of starter LAB during cheese production.

Samples	Bacterial Counts	
	TMM	*Lc. lactis*
Curd		
CP	7.89 ± 0.25	7.99 ± 0.33
EPL100	8.01 ± 0.21	8.11 ± 0.20
EPL200	7.78 ± 0.27	7.90 ± 0.25
EPO100	8.12 ± 0.20	8.15 ± 0.25
EPO200	7.97 ± 0.18	8.01 ± 0.31
EPT100	7.71 ± 0.26	8.07 ± 0.30
EPT200	7.83 ± 0.23	8.03 ± 0.23
*p* value	0.392	0.937
Cheese		
CP	9.03 ± 0.35	9.12 ± 0.29
EPL100	9.01 ± 0.27	9.19± 0.24
EPL200	9.33 ± 0.21	9.38 ± 0.23
EPO100	9.15 ± 0.24	9.35 ± 0.30
EPO200	9.20 ± 0.21	9.33 ± 0.27
EPT100	9.14 ± 0.20	9.24 ± 0.24
EPT200	9.04 ± 0.30	9.20 ± 0.31
*p* value	0.742	0.878

Units are Log CFU/g. Results indicate the mean values and standard deviation of four plate counts (carried out in duplicate for two independent productions). Abbreviations: TMM, total mesophilic microorganisms; *Lc.*, *Lactococcus*; CP, control production inoculated with Natural Milk Starter Cultures (NMSC); EPL100, experimental production inoculated with NMSC + 100 µL/L of lemon essential oils (EOs); EPL200, experimental production inoculated with NMSC + 200 µL/L of lemon EOs; EPO100, experimental production inoculated with NMSC + 100 µL/L of orange EOs; EPO200, experimental production inoculated with NMSC + 200 µL/L of orange EOs; EPT100, experimental production inoculated with NMSC + 100 µL/L of tangerine EOs; EPT200, experimental production inoculated with NMSC + 200 µL/L of tangerine EOs.

**Table 3:** Physicochemical traits of Primosale cheeses.

	Samples							SEM	*p*-Value
	CP	EPL100	EPL200	EPO100	EPO200	EPT100	EPT200		
External color									
Lightness, L*	79.39 abc	77.32 bc	76.38 c	82.12 a	81.61 a	79.70 ab	77.07 bc	0.56	0.0009
Redness, a*	−2.23 a	−4.12 cd	−4.51 d	−3.21 b	−3.46 bc	−4.34 d	−4.15 cd	0.15	0.0001
Yellowness, b*	17.75 a	16.69 ab	16.52 ab	12.65 c	14.75 bc	14.66 bc	14.77 abc	0.53	0.0032
Internal color									
Lightness, L*	85.81 a	80.23 b	80.94 b	87.31 a	85.76 a	77.96 b	76.97 b	0.71	<0.0001
Redness, a*	−3.54 abc	−4.36 cd	−4.29 bcd	−3.18 a	−3.29 ab	−4.93 d	−4.85 d	0.18	0.0010
Yellowness, b*	13.36 ab	13.61 ab	14.65 a	12.23 b	12.44 b	14.49 a	13.57 ab	0.34	0.0092
Hardness, N/mm^2^	0.63 ab	0.56 b	0.58 ab	0.75 a	0.58 ab	0.51 b	0.57 ab	0.032	0.0194
Water activity, a_w_	0.97	0.98	0.98	0.97	0.96	0.98	0.99	0.028	0.9970
Dry matter (DM), %	57.05	59.72	57.76	58.47	59.70	57.36	59.27	1.69	0.8428
Fat, % DM	45.90	42.25	43.27	42.81	44.45	43.48	45.01	1.27	0.4417
Protein, % DM	46.91	50.51	49.29	49.54	47.42	48.91	48.30	1.48	0.6457
Ash, % DM	7.19 ab	7.24 ab	7.45 ab	7.65 ab	8.13 a	7.61 ab	6.69 b	0.21	0.0106

Results indicate the mean values of determinations carried out in duplicate for each of the two independent productions. Data within a row followed by different letters are significantly different according to Tukey’s test (*p* < 0.05). Abbreviations: SEM = standard error of the mean; CP, control production inoculated with the Natural Milk Starter Cultures (NMSC); EPL100, experimental production inoculated with NMSC + 100 µL/L of lemon essential oils (EOs); EPL200, experimental production inoculated with NMSC + 200 µL/L of lemon EOs; EPO100, experimental production inoculated with NMSC + 100 µL/L of orange EOs; EPO200, experimental production inoculated with NMSC + 200 µL/L of orange EOs; EPT100, experimental production inoculated with NMSC + 100 µL/L of tangerine EOs; EPT200, experimental production inoculated with NMSC + 200 µL/L of tangerine EOs.

**Table 4:** Antioxidant capacity of the Primosale cheeses.

Samples	Polyphenols (g GAE/kg DM)	TEAC (mmol/kg DM)	POV (mEq O_2_/kg fat)	TBARS (mg MDA/kg DM)
CP	5.45	40.66 b	2.20 bc	3.07 e
EPL100	5.99	63.82 a	2.09 d	4.75 d
EPL200	6.13	59.56 a	2.07 d	4.07 d
EPO100	5.47	61.88 a	2.15 c	7.73 b
EPO200	5.84	59.41 a	2.25 a	4.24 d
EPT100	5.90	59.05 a	2.19 bc	10.23 a
EPT200	6.60	60.13 a	2.21 ab	5.99 c
SEM	0.49	4.46	0.011	0.16
*p*-value	0.6883	0.0179	<0.0001	<0.0001

Results indicate the mean values of the determinations carried out in duplicate for each of the two independent productions. Data within a column followed by different letters are significantly different according to Tukey’s test (*p* < 0.05). Abbreviations: CP, control production inoculated with the Natural Milk Starter Cultures (NMSC); EPL100, experimental production inoculated with NMSC + 100 µL/L of lemon essential oils (EOs); EPL200, experimental production inoculated with NMSC + 200 µL/L of lemon EOs; EPO100, experimental production inoculated with NMSC + 100 µL/L of orange EOs; EPO200, experimental production inoculated with NMSC + 200 µL/L of orange EOs; EPT100, experimental production inoculated with NMSC + 100 µL/L of tangerine EOs; EPT200, experimental production inoculated with NMSC + 200 µL/L of tangerine EOs; SEM = standard error of mean; GAE, gallic acid equivalent; DM, dry matter; TEAC, Trolox equivalent antioxidant capacity; POV, peroxide value; TBARS, thiobarbituric acid-reactive substances; MDA, malonylaldehyde.

**Table 5:** Volatile organic compounds emitted Primosale cheeses.

Chemical Compounds	Samples							*p* Value
	CP	EPL100	EPL200	EPO100	EPO200	EPT100	EPT200	
Acids								
Acetic acid	3.73 ± 0.74 a	0.38 ± 0.07 b	0.28 ± 0.07 b	0.41 ± 0.07 b	0.28 ± 0.0 b	0.40 ± 0.08 b	0.31 ± 0.08 b	<0.0001
Butanoic acid	7.96 ± 1.42 a	1.49 ± 0.23 b	1.29 ± 0.32 b	1.47 ± 0.29 b	1.18 ± 0.29 b	1.67 ± 0.30 b	1.37 ± 0.35 b	<0.0001
Exanoinc acid	14.77 ± 2.77 a	3.00 ± 0.61 b	2.42 ± 0.57 b	2.44 ± 0.52 b	2.05 ± 0.51 b	3.18 ± 0.69 b	2.66 ± 0.67 b	<0.0001
Pentanoinc acid-2-hydroxy-4-methyl	9.99 ± 2.12 a	1.43 ± 0.26 b	1.2 ± 0.29 b	1.21 ± 0.32 b	0.99 ± 0.25 b	1.37 ± 0.28 b	1.09 ± 0.25 b	<0.0001
Octanoic Acid	5.13 ± 0.76 a	1.14 ± 0.22 b	0.99 ± 0.25 bc	0.74 ± 0.13 bc	0.17 ± 0.04 c	0.99 ± 0.19 bc	0.77 ± 0.18 bc	<0.0001
Nonanoic Acid	0.83 ± 0.14 a	n.d. b	n.d. b	n.d. b	n.d. b	n.d. b	n.d. b	<0.0001
Ketons								
2-pentanone	1.34 ± 0.26 a	0.15 ± 0.03 b	0.010 ± 0.002 b	0.11 ± 0.02 b	0.05 ± 0.01 b	0.07 ± 0.01 b	0.06 ± 0.01 b	<0.0001
3-hydroxy-2-butanone	0.06 ± 0.01 a	n.d. b	n.d. b	n.d. b	n.d. b	n.d. b	n.d. b	<0.0001
3,5 octadien-2-one	0.39 ± 0.07 a	0.20 ± 0.03 b	0.12 ± 0.02 bcd	0.12 ± 0.02 bcd	0.03 ± 0.01 d	0.10 ± 0.01 cd	0.14 ± 0.03 bc	<0.0001
Alcohol								
1-butanol-3-methyl	7.02 ± 1.41 a	1.24 ± 0.38 b	0.99 ± 0.24 b	0.90 ± 0.23 b	0.72 ± 0.18 b	1.27 ± 0.29 b	1.02 ± 0.25 b	<0.0001
1 pentanol	0.81 ± 0.11 a	0.10 ± 0.0 b	0.07 ± 0.01 b	0.06 ± 0.01 b	0.03 ± 0.01 b	0.06 ± 0.01 b	0.04 ± 0.01 b	<0.0001
4 methyl-1-pentanol	n.d. e	0.09 ± 0.01 a	0.06 ± 0.01 bc	0.07 ± 0.01 ab	0.04 ± 0.01 cd	0.03 ± 0.01 d	0.02 ± 0.01 de	<0.0001
Octan-1-ol	2.79 ± 0.46 a	0.78 ± 0.13 bc	0.05 ± 0.01 d	1.09 ± 0.19 b	0.45 ± 0.10 cd	0.59 ± 0.1 bcd	0.20 ± 0.05 d	<0.0001
Aldeyde								
2-pentenal	0.65 ± 0.09 a	0.08 ± 0.01 b	0.03 ± 0.01 b	0.07 ± 0.01 b	0.03 ± 0.01 b	0.05 ± 0.01 b	0.04 ± 0.01 b	<0.0001
Hexanal	16.02 ± 2.01 a	2.73 ± 0.50 b	2.01 ± 0.48 b	1.77 ± 0.32 b	1.46 ± 0.38 b	1.70 ± 0.40 b	1.37 ± 0.34 b	<0.0001
2-butanal	0.18 ± 0.03 a	0.20 ± 0.04 a	0.11 ± 0.027 bc	0.17 ± 0.03 ab	0.04 ± 0.01 d	0.07 ± 0.01 cd	0.010 ± 0.002 d	<0.0001
Heptanal	17.72 ± 3.60 a	3.63 ± 0.64 b	2.90 ± 0.68 b	3.24 ± 0.75 b	2.51 ± 0.65 b	2.79 ± 0.51 b	2.31 ± 0.6 b	<0.0001
Nonanal	1.83 ± 0.36 a	n.d. b	n.d. b	n.d. b	n.d. b	n.d. b	n.d. b	<0.0001
2-octenal	1.74 ± 0.37 a	n.d. b	n.d. b	n.d. b	n.d. b	n.d. b	n.d. b	<0.0001
2-nonenal	5.27 ± 0.98 a	0.91 ± 0.12 b	0.79 ± 0.19 b	0.84 ± 0.14 b	0.54 ± 0.13 b	0.22 ± 0.03 b	0.19 ± 0.04 b	<0.0001
Monoterpene Hydrocarbons								
α-pinene	1.78 ± 0.29 a	0.76 ± 0.14 b	0.47 ± 0.12 bc	0.20 ± 0.04 c	0.17 ± 0.04 c	0.71 ± 0.14 b	0.50 ± 0.13 bc	<0.0001
β-pinene	n.d. d	0.11 ± 0.02 c	0.07 ± 0.01 cd	0.24 ± 0.04 b	0.09 ± 0.01 cd	0.38 ± 0.06 a	0.33 ± 0.07 ab	<0.0001
Myrcene	n.d. c	0.06 ± 0.01 c	0.020 ± 0.004 c	2.04 ± 0.42 ab	1.99 ± 0.51 b	3.05 ± 0.63 a	1.84 ± 0.42 b	<0.0001
Phellandrene	n.d. b	0.040 ± 0.008 a	0.03 ± 0.01 a	n.d. b	n.d. b	n.d. b	n.d. b	<0.0001
Limonene	n.d. b	77.99 ± 3.96 a	84.2 ± 3.36 a	80.79 ± 3.79 a	86.09 ± 3.44 a	79.39 ± 3.49 a	85.18 ± 4.26 a	<0.0001
Carene	n.d. d	2.70 ± 0.47 a	1.99 ± 0.43 ab	0.07 ± 0.01 d	0.020 ± 0.005 d	1.29 ± 0.28 bc	0.99 ± 0.22 c	<0.0001
Oxygenated monoterpenes								
Linalool	n.d. c	0.42 ± 0.07 c	0.32 ± 0.07 c	1.83 ± 0.37 a	1.06 ± 0.24 b	0.37 ± 0.07 c	0.10 ± 0.02 c	<0.0001
α -Terpineol	n.d. d	0.39 ± 0.08 a	0.10 ± 0.02 cd	0.12 ± 0.02 c	0.05 ± 0.01 cd	0.25 ± 0.05 b	0.09 ± 0.02 cd	<0.0001

Results indicate the mean percentage values of three measurements and are expressed as relative peak areas (peak area of each compound/total area of the significant peaks in all samples) × l00. Data within a row followed by different letters are significantly different, according to Tukey’s test (*p* < 0.05). Abbreviations: CP, control production inoculated with the Natural Milk Starter Cultures (NMSC); EPL100, experimental production inoculated with NMSC + 100 µL/L of lemon essential oils (EOs); n.d., not detectable.

**Table 6:** Evaluation of the sensory attributes of Primosale cheeses.

Attributes	Trial							SEM	*p*-Value	
	CP	EPL100	EPL200	EPO100	EPO200	EPT100	EPT200		Judges	Cheeses
Color	3.87	3.95	4.05	3.85	3.95	3.91	4.03	0.06	0.929	0.962
Structure uniformity	5.41	5.25	5.33	5.26	5.27	5.36	5.45	0.06	0.536	0.967
Intensity of odor	4.88 c	5.38 abc	5.85 ab	5.29 bc	5.67 ab	5.62 ab	5.96 a	0.07	0.179	<0.0001
Unpleasant odor	0.00	0.00	0.00	0.00	0.00	0.00	0.00	0.00	1.00	1.00
Intensity of aroma	5.19 d	5.59 bdc	6.05 ab	5.46 cd	5.95 abc	5.67 bcd	6.25 a	0.06	0.259	<0.0001
Sweet	5.24	5.19	5.10	5.22	5.15	5.05	5.01	0.05	0.052	0.826
Salty	3.54	3.42	3.48	3.56	3.55	3.46	3.50	0.03	0.444	0.933
Acid	2.39	2.61	2.81	2.48	2.84	2.74	2.92	0.06	0.068	0.092
Bitter	1.51	1.49	1.62	1.57	1.65	1.65	1.59	0.04	0.435	0.895
Spicy	1.93	1.81	1.94	2.05	2.06	1.91	2.05	0.05	0.155	0.828
Adhesiveness	2.53 ab	2.35 b	2.65 ab	2.87 a	2.69 ab	2.66 ab	2.82 ab	0.05	0.213	0.062
Hardness	3.93	3.65	3.62	3.88	3.72	3.51	3.83	0.06	0.586	0.376
Humidity	2.81	2.59	2.52	2.37	2.47	2.77	2.41	0.05	0.244	0.053
Taste persistency	3.15 c	3.49 bc	3.98 a	3.31 c	3.79 ab	3.55 bc	4.16 a	0.06	0.969	<0.0001
Unpleasant aroma	0.00	0.00	0.00	0.00	0.00	0.00	0.00	0.00	1.00	1.00
Overall acceptability	4.73 c	6.05 a	4.27 c	5.43 b	4.09 c	5.69 ab	4.21 c	0.10	0.998	<0.0001

Results indicate the mean value. Data within a row followed by the same letter are not significantly different according to Tukey’s test. Abbreviations: CP, control production inoculated with the Natural Milk Starter Cultures (NMSC); EPL100, experimental production inoculated with NMSC + 100 µL/L of lemon essential oils (EOs); EPL200, experimental production inoculated with NMSC + 200 µL/L of lemon EOs; EPO100, experimental production inoculated with NMSC + 100 µL/L of orange EOs; EPO200, experimental production inoculated with NMSC + 200 µL/L of orange EOs; EPT100, experimental production inoculated with NMSC + 100 µL/L of tangerine EOs; EPT200, experimental production inoculated with NMSC + 200 µL/L of tangerine EOs.

## Data Availability

All data included in this study are available upon request by contacting the corresponding author.
